# Peripheral B cell immune dysregulation genetically contributes to stage-dependent neuroinflammation and identifies priority therapeutic targets in Parkinson’s disease: a computational integration of Mendelian randomization and single-cell transcriptomics

**DOI:** 10.3389/fmed.2026.1853077

**Published:** 2026-06-09

**Authors:** Lu Zhang, Chengwei Fu, Ting Gao, Kezhen Yang, Gang Ouyang, Wenjun Chen

**Affiliations:** 1 Department of Rehabilitation Medicine, Sir Run Run Shaw Hospital, Zhejiang University School of Medicine, Hangzhou, China; 2 Department of Acupuncture, Hubei Provincial Hospital of Traditional Chinese Medicine, Wuhan, China; 3 Department of Acupuncture, Geriatric Hospital of Nanjing Medical University, Nanjing, China

**Keywords:** B cells, drug repurposing, intercellular communication, Mendelian randomization, neuroinflammation, Parkinson’s disease, peripheral immunity, single-cell eQTL

## Abstract

**Background:**

Parkinson’s disease (PD) lacks reliable peripheral biomarkers and disease-modifying therapies. Although peripheral B-cell alterations have been observed in PD, whether genetically predicted B-cell gene expression contributes to PD risk and can nominate candidate therapeutic targets remains unclear.

**Methods:**

We established a computational framework integrating two-sample Mendelian randomization (MR) based on B-cell subtype-specific sc-eQTLs (OneK1K) and PD GWAS (IPDGC; 33,674 cases/449,056 controls), Bayesian colocalization with DICE and FinnGen replication, exploratory pseudobulk stage-associated analysis (GSE223138), B-cell subpopulation fine-mapping in 10,466 CD19 + cells (GSE194245), CellChat v2 communication inference, and druggability prioritization with molecular docking-based structural plausibility assessment.

**Results:**

Multi-layer computational evidence prioritized eight high-confidence MR causal genes: five risk-increasing genes (CD74, HLA-DRB1, IL2RA, BLK, BANK1) and three protective genes (CR1, PTPN22, FCRL3). Exploratory pseudobulk analysis suggested four tentative stage-associated expression patterns, with early-change genes (CD74, HLA-DRB1, IL2RA) showing expression differences detectable as early as PD-Early in a small exploratory cohort. Subpopulation analysis suggested enrichment in Naive B (BLK, BANK1) and Atypical B cells (CD74, HLA-DRB1, IL2RA, CR1), while acknowledging that these fine-grained assignments are not equivalent to MR exposure categories. CellChat analysis predicted that the MIF signaling pathway showed an inferred communication probability increasing from 0.15 in HC to 0.48 in PD-Late. Five-dimensional evidence convergence nominated CD74, IL2RA, and CR1 as candidate targets, with molecular docking suggesting structural plausibility for selected small-molecule interactions, including Dasatinib-BLK (−7.2 kcal/mol) and ISO-1-CD74 (−6.8 kcal/mol).

**Conclusion:**

These computational findings suggest that genetically influenced B-cell gene expression may contribute to peripheral immune dysregulation in PD and nominate CD74, IL2RA, and CR1 as candidate targets for future validation. The results should be interpreted as hypothesis-generating and require experimental and clinical confirmation.

## 1 Introduction

Parkinson’s disease (PD) is the second most common neurodegenerative condition worldwide. PD’s defining pathology is a constant reduction of dopaminergic neurons from the substantia nigra and the accumulation of Lewy bodies. More than 90 loci of genetic factors affecting PD have been identified, thereby emphasizing the intricate nature of the disorder (1). Previous studies have traditionally focused on the pathological mechanisms in the central nervous system (CNS), while recent evidence has accumulated to indicate that the peripheral immune system is playing an indispensable role in the pathogenesis of PD. The level of pro-inflammatory cytokines in peripheral blood in PD patients is increased. The immune cell composition is significantly altered in PD patients. The incidence of autoimmune diseases in patients with PD is significantly increased (2). With regard to neuroinflammation, the innate immune response mediated by microglia and astrocytes in the CNS is widely regarded as a common pathological feature among neurodegenerative diseases (3). The mechanisms by which peripheral adaptive immune cells are involved indirectly in neuroinflammation, such as through the migration of a damaged blood–brain barrier to the CNS, substantial changes to the composition of peripheral immune cells, and activation of the complement system through the body fluid pathway, have also been gradually revealed (4). Though the single-cell RNA sequencing technique has achieved unprecedented resolution in the systematic mapping of the peripheral immune landscape in PD patients, the fundamental causality issue of whether the observed changes in the immune cells are the causative factors of PD or just the consequences of the disease process itself has not been addressed, and the genetic basis of the interaction of the peripheral and central immune system has not been clarified (5). In the research of the peripheral immune in PD, the potential role of B cells has gradually been emphasized, and their roles might be far beyond the classic single dimension of antibody production. The pro-inflammatory and neurotoxic roles of innate immune cells such as natural killer cells in PD have been clearly elucidated, while the roles of B cells, as the main effector cells of the adaptive immune system, have been behind in the research of PD but have developed rapidly (6). Peripheral blood scRNA-seq combined with B cell receptor (BCR) sequencing has revealed phenotypic characteristics such as increased memory B cells, decreased naive B cells, and enhanced IgG/IgA isotype conversion in PD patients, suggesting that B cells have undergone abnormal activation and differentiation processes in PD patients (7). At the same time, the level of *α*-synuclein-specific autoantibodies has a declining trend in the prodromal stage of PD. However, the reliability of the level as a PD biomarker is limited by a large inter-queue variation and differences in methods (8, 9). These contradictory findings exactly reflect the lack of understanding of the function of B cells in PD. It is worth noting that there are three systematic gaps in the research on B cells and PD: All the current studies are descriptive association studies and cannot establish a causality relationship between B cell changes and the risk of PD; There is a lack of dynamic analysis with a dimension of the stage of the disease; Whether the changes in B cells are an immune phenomenon occurring early in the course of the disease or a consequence of late neurodegeneration is unknown; There is a lack of a systematic link between B cell causality discovery and drug targets.

Mendelian randomization has the potential to offer an important genetic epidemiological approach to address the aforementioned challenges in causal inference. This method of using genetic variation as an instrumental variable has the ability to exclude confounding factors and reverse causality bias, as seen in traditional observational studies, and offers an assured framework of inference on the causal pathway from gene expression to disease risk. The development of large-scale single-cell expression quantitative trait loci has made it possible to conduct cell type-specific MR analysis. OneK1K has established the genetic regulatory map of cell types, comprising 1.27 million peripheral blood mononuclear cells from 982 healthy individuals and has identified the cis-eQTL at the B cell subtype level as an instrumental variable (10). Meanwhile, eQTL research based on single-cell data has further validated that the regulatory effect of genetic variation on gene expression is significantly dependent on cell types, and this cell-type specificity and disease state dependency of eQTL effect is of central importance in the genetic basis analysis of immune-mediated diseases (11, 12). By integrating MR causal screening with scRNA-seq single-cell data validation and further employing Bayesian co-localization analysis to differentiate true positive effect from false positive effect induced by linkage disequilibrium, cross-level integration can be realized from genetic causal discovery to single-cell data mechanism analysis. The existing integration paradigms were mainly focused on extensive screening of various cell types; however, deficiencies exist in the in-depth analysis dimensions in terms of localization of single cell types’ subgroups and state transition trajectories in disease stage dynamics.

Recent MR studies have begun to explore the causal relationship between the characteristics of peripheral immune cells and the risk of PD (13, 14). Some studies have further conducted bidirectional causal tests between immune cell phenotypes and PD (15). However, in these studies, B cells usually remained at the level of broad immune-cell screening. The specific localization of subpopulations, disease-stage-associated expression patterns, and B cell-specific communication remodeling have remained insufficiently characterized. Therefore, this study constructs a computational framework based on public datasets to integrate B-cell subtype sc-eQTL-based MR, colocalization, single-cell transcriptomic validation, CellChat-based communication inference, and druggability analysis. By design, this framework generates testable hypotheses rather than definitive experimental proof, and all prioritized genes, pathways, and candidate compounds require future biological and clinical validation.

## 2 Materials and methods

### 2.1 Data sources and preprocessing

The MR analysis of the exposure data was based on the B cell sc-eQTL of the OneK1K cohort. The OneK1K cohort carried out scRNA-seq on 1,267,758 peripheral blood mononuclear cells (PBMCs) from 982 healthy individuals and identified cis-eQTLs for 14 immune cell types. The B cell section included independent eQTLs for the two subtypes of initial B cells, B Immature/Naive (BIN), and memory B cells, BMem, which were publicly available at onek1k.org. The primary consideration for choosing OneK1K over eQTLGen was that the former provides cell type-specific eQTL, enabling the distinction of gene regulatory effects at the B cell subtype level, while the latter is a bulk eQTL of whole blood and cannot achieve cell type discrimination. Independent replication was conducted using the B cell eQTL from the DICE database (91 healthy donors, dice-database.org), with a replication standard of consistent effect direction and *p* < 0.05. Outcome data utilized the PD GWAS summary statistics published by IPDGC [(16); 33,674 cases, 449,056 controls, European population; available at https://pdgenetics.org], and a sensitivity analysis was performed using the FinnGen PD GWAS for independent replication.

The scRNA-seq data include a discovery set and a validation set with complementary designs. The discovery set GSE223138 contains 6 PBMC samples (2 healthy controls, 2 in early PD stage, and 2 in late PD stage), sequenced using the 10 × Genomics platform. The quality control criteria are a mitochondrial gene proportion of less than 10%, a gene count of 300–7,500, and a unique molecular identifier (UMI) count of no less than 600. After LogNormalize standardization, 2,000 variable genes screening, principal component analysis (PCA) dimension reduction, and Harmony batch correction, clustering was performed using clustree with optimized resolution, Uniform Manifold Approximation and Projection (UMAP) visualization, and cell annotation was carried out using a dual verification strategy of classic marker genes and SingleR. The B cell cluster was extracted for downstream analysis. The proportion of B cells in each sample was calculated as the number of B cells divided by the total number of PBMCs after quality control. The validation set GSE194245 contains 10,466 CD19^+^ fluorescence-activated cell sorting (FACS)-sorted B cells (8 cases of PD and 6 cases of healthy controls), and the original count matrix was independently downloaded and processed according to the same procedure.

The research only utilized public aggregate data from existing statistical data and public scRNA-seq data from the GEO database. Note that all original data have been pre-approved with informed consent in their original studies. No additional approval is necessary. Note that the causal effects of MR refer to the genetically predicted expression levels of genes on PD risk, and the expression differences between cases and controls at the single-cell level refer to disease-associated transcriptomic changes. They are conceptually different from each other. The single-cell data presented in this research are intended to offer clues for biological plausibility of MR-identified causal genes.

The datasets used in this study were generated from different cohorts, laboratories, and platforms and were therefore not treated as a unified experimental multi-omics cohort. Instead, they were integrated as complementary public resources: MR and colocalization provided genetic evidence, whereas the scRNA-seq datasets provided transcriptomic and cell-state evidence. This design supports cross-dataset hypothesis generation but also introduces technical and biological heterogeneity that should be considered when interpreting the results.

### 2.2 Two-sample Mendelian randomization analysis

This study was conducted following the STROBE-MR reporting guideline for Mendelian randomization studies (17). The MR analysis followed current standardized recommendations and conducted two-sample MR for both BIN and BMem separately (18). The instrumental variables were selected from the OneK1K B-cell sc-eQTL, covering 1 Mb upstream and downstream of each gene with *p* < 5 × 10^−8^ for cis-eQTL. The linkage disequilibrium (LD) pruning threshold was set at r^2^ < 0.001, with a window size of 10,000 kb, using the European population from 1,000 Genomes as the reference panel. Single nucleotide polymorphisms with F-statistics greater than 10 were retained to reduce weak-instrument bias.

Cochran’s Q test for heterogeneity, MR-Egger intercept test, MR-PRESSO, and leave-one-out analysis were used as sensitivity analyses. In reverse MR, PD GWAS loci were considered as exposures and B-cell gene expression as the outcome. Non-significant reverse MR results were interpreted as the absence of strong evidence for reverse causation rather than definitive proof of a unidirectional causal relationship, because reverse MR using gene expression as the outcome may have limited statistical power relative to the forward GWAS-based analysis. FinnGen and DICE were used for validation in outcomes and exposures, respectively. Bayesian colocalization was performed using the coloc R package within 500 kb windows around each significant locus, and PP. H4 > 0.80 was considered evidence supporting shared causal variants between eQTL and PD GWAS signals. Although an alternative instrument-selection threshold (e.g., *p* < 5 × 10^−6^) was not formally tested, the concordance among three MR methods (IVW, MR-Egger, and weighted median) for all eight prioritized genes provides indirect evidence of robustness to instrument selection.

### 2.3 Validation of the temporal dynamics of causal genes

B-cell clusters were extracted from GSE223138. Because each group contained only two biological replicates, all stage-associated analyses were treated as exploratory and hypothesis-generating. To reduce pseudo-replication, the B-cell expression matrices were aggregated into sample-level pseudobulk profiles (19). This strategy was selected because cells from the same individual are not statistically independent, and pseudobulk approaches reduce pseudoreplication bias by preserving the biological sample as the unit of inference (20, 21). Based on the sample as the biological unit, DESeq2 was used for pairwise differential expression analysis among HC, PD-Early, and PD-Late groups. The stage-associated classification was used to describe tentative expression patterns rather than to establish definitive temporal trajectories.

### 2.4 Fine localization and state transition analysis of B cell subpopulations

For GSE194245, all 14 donors (8 PD, 6 HC) passed quality control, yielding a total of 10,466 B cells that were independently clustered and annotated into subpopulations based on classical marker genes. It should be noted that the MR exposure categories from OneK1K (BIN and BMem) are broad B-cell categories and do not map one-to-one to the six B-cell subpopulations identified in GSE194245. Biologically, BIN broadly corresponds to early B-cell states such as Naive B and Transitional B cells, whereas BMem broadly corresponds to memory-like states such as Memory B and Atypical B cells. Plasma B and Proliferating B represent more terminal or cycling states that are less directly captured by either MR category. Therefore, subpopulation localization was used to explore biological plausibility at higher resolution rather than to assign MR-inferred causal effects definitively to individual B-cell states.

### 2.5 Functional enrichment and analysis of immune inflammation pathways

Gene Ontology (GO)/Kyoto Encyclopedia of Genes and Genomes (KEGG) enrichment analyses were performed on the causal genes. Immune-inflammatory pathway activity among B-cell subpopulations was evaluated using Gene Set Variation Analysis (GSVA), and the PPI network of causal genes, PD risk genes, and inflammatory mediators was constructed by combining STRING with Cytoscape. For transparency, the genes contributing to the main GO/KEGG and Hallmark pathway annotations were reported in the text and summarized in Supplementary Table S3.

### 2.6 Analysis of the peripheral immune inflammatory communication network of B cells

CellChat v2 was used to infer intercellular communication from the entire PBMC dataset of GSE223138, focusing on communication networks between B cells and monocytes, NK cells, and T cells (22). CellChat was selected because benchmark studies have shown that ligand-receptor-based communication predictions vary across tools and resources, while CellChat performs favorably in several comparative evaluations, including analyses incorporating spatial information (23, 24). CellChat estimates communication probabilities from gene expression and curated ligand-receptor interactions; therefore, these results were interpreted as computational predictions rather than experimentally measured interactions. The analysis compared communication intensity among the three groups and screened immune-inflammatory ligand-receptor pairs, including MIF-CD74, CXCL-CXCR, and complement-related interactions. It also examined whether MR-prioritized genes encode known communication molecules. Because only one communication-inference framework was used, the results require future validation with complementary tools and orthogonal experimental evidence.

### 2.7 Prediction of druggable candidate targets and molecular docking analysis

The causal genes supported by MR and colocalization were screened as potential druggable targets using DGIdb, and candidate repurposing compounds were prioritized using DSigDB. This analysis was designed to evaluate whether existing compounds with known pharmacological annotations may be considered for future PD-related immunomodulatory studies, rather than to rediscover known drug-target binding. Molecular docking was restricted to selected small-molecule or peptide-like candidate interactions for which structural plausibility could be evaluated. Crystal structures of candidate targets were obtained from PDB (resolution < 2.5 Å), and AutoDock Vina was used to estimate binding free energy after preprocessing with PyMOL. Docking scores were interpreted as structural plausibility evidence only and not as pharmacological validation. Cytoscape constructed an integrated network of causal genes, druggability annotations, candidate compounds, and multi-dimensional evidence convergence.

## 3 Results

### 3.1 Mendelian randomization for the identification of B-cell-specific causal genes for Parkinson’s disease

**Figure 1:**
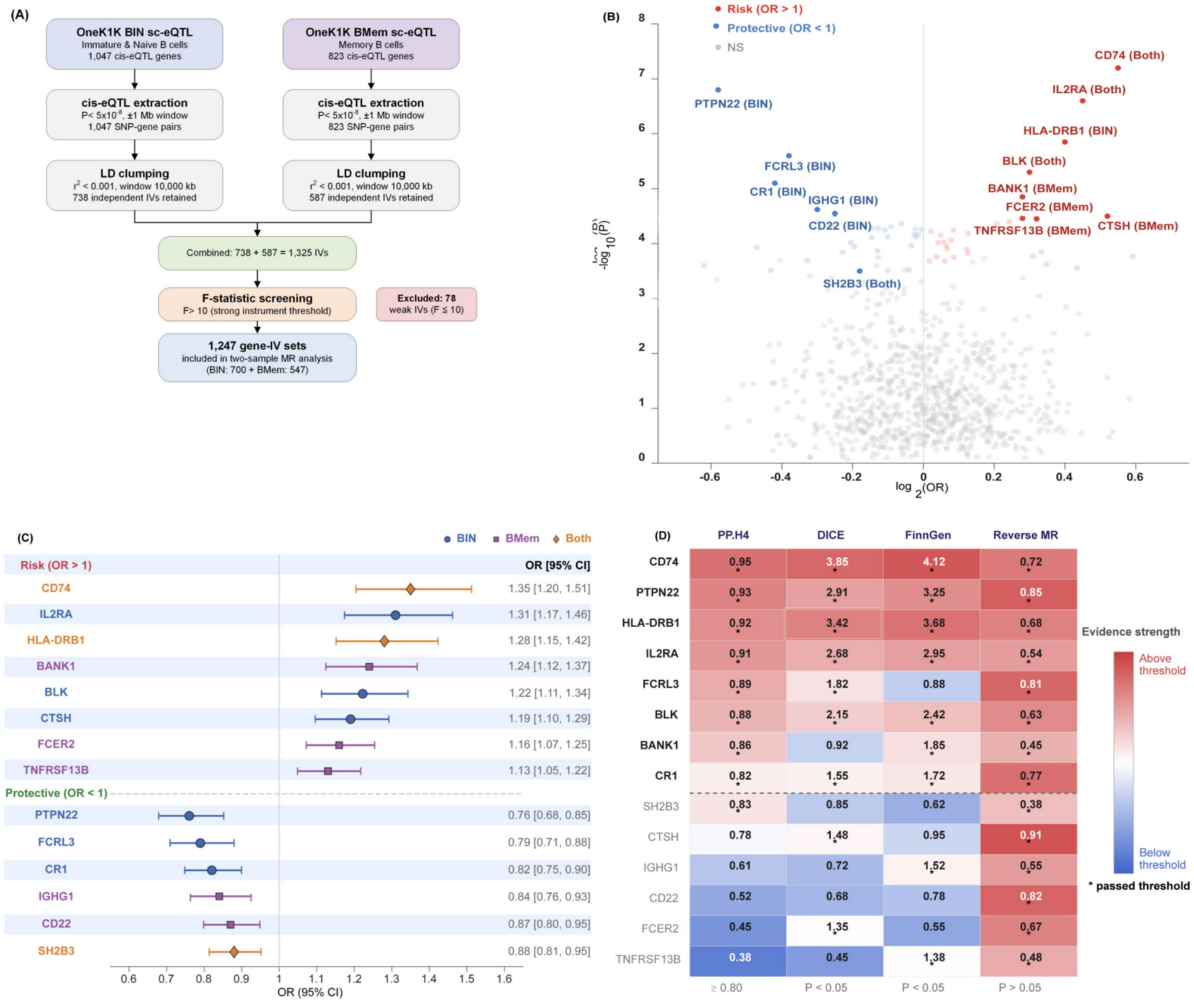
Mendelian randomization identification of B-cell-specific genes associated with Parkinson’s disease risk. **(A)** Flowchart of instrumental variable selection from OneK1K BIN and BMem sc-eQTL data. **(B)** Volcano plot of MR screening results, with genes labeled by B-cell subtype origin (BIN, BMem, or Both). **(C)** Forest plot of IVW effect estimates (OR and 95% CI) for 14 MR-prioritized genes, grouped by risk-increasing (OR > 1) and protective (OR < 1) effects. **(D)** Summary heatmap of multi-layer computational evidence. The PP. H4 column displays colocalization posterior probabilities (threshold ≥ 0.80); the DICE and FinnGen columns display −log₁₀(P) from independent replication analyses (threshold: −log_10_(0.05) = 1.30, corresponding to *p* < 0.05); the reverse MR column displays −log_10_(*P*) from the reverse MR IVW analysis, where lower values (blue) indicate non-significant reverse effects (*p* > 0.05), consistent with a forward causal direction. Reverse MR findings should be interpreted in light of limited statistical power when gene expression is the outcome. Raw *p*-values for all analyses are reported in Supplementary Table S1. Asterisks indicate values passing the respective threshold.

According to the sc-eQTL information of two kinds of B cells, BIN and BMem, of OneK1K, there are 1,047 and 823 cis-eQTL genes, respectively. After LD thinning and F statistic screening, a total of 1,247 gene instrumental variables are selected for two-sample MR analysis (Figure 1A).

The MR screening results were presented in the form of volcano plots, and significant genes after multiple-testing correction were labeled according to BIN and BMem sources (Figure 1B). The IVW principal analysis identified 14 B-cell genes associated with PD risk after Benjamini-Hochberg FDR correction (FDR < 0.05) (Figure 1C), of which 6 also passed the more stringent Bonferroni threshold (*p* < 0.05/1,247 = 4.01 × 10^−5^). The Bonferroni-significant genes included BANK1 (*p* = 3.5 × 10^−5^), meeting the corrected threshold. For downstream analyses, Bonferroni significance was reported as an additional stringent benchmark rather than used as a mandatory inclusion criterion. We applied triple validation criteria requiring: (1) FDR significance in the IVW analysis, (2) strong colocalization evidence (PP. H4 > 0.80), and (3) at least one independent replication signal in DICE or FinnGen with a consistent effect direction. Eight genes met all three criteria simultaneously, including two genes (BLK and CR1) that passed FDR correction but not Bonferroni correction.

In the multi-layer verification stage, the colocalization posterior probabilities, DICE validation status, FinnGen validation status, and reverse MR results of all 14 FDR-significant genes were integrated and presented as a summary heatmap (Figure 1D). Colocalization analysis revealed evidence of shared eQTL and PD GWAS signals for 9 genes (PP. H4 > 0.80). The DICE B-cell eQTL independent cohort supported the consistency of effect directions for seven genes (*p* < 0.05), and FinnGen supported replication for several prioritized genes. Reverse MR did not detect significant reverse effects, which is consistent with the forward MR findings. However, given the lower power of reverse MR when gene expression is modeled as the outcome, these results should not be interpreted as definitive evidence excluding modest reverse causation. Based on FDR significance, colocalization, and independent replication, eight high-confidence MR causal genes were prioritized for downstream computational analyses. Full MR estimates and sensitivity analyses are shown in Supplementary Table S1 and Supplementary Figure S1.

### 3.2 Disease stage-associated expression changes of causal genes in Parkinson’s disease

**Figure 2:**
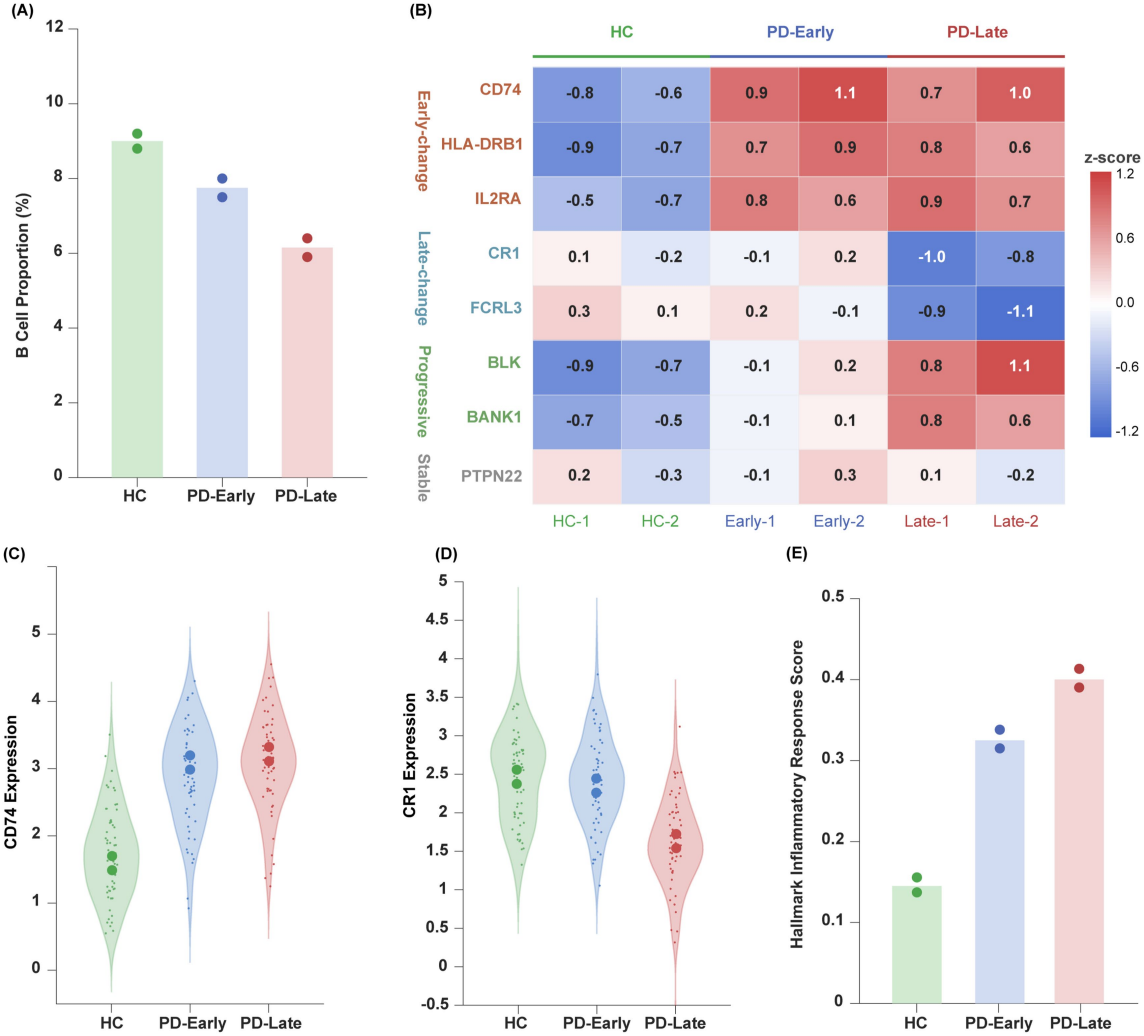
Exploratory disease stage-associated expression changes of causal genes in Parkinson’s disease. **(A)** B-cell proportion across HC, PD-Early, and PD-Late groups in GSE223138. **(B)** Pseudobulk expression heatmap of 8 high-confidence MR causal genes across 6 samples, with rows clustered by tentative expression pattern (early-change, late-change, progressive, stable) and columns ordered by disease stage. **(C)** Single-cell expression distribution and pseudobulk mean of CD74 across three groups. **(D)** Single-cell expression distribution and pseudobulk mean of CR1 across three groups. **(E)** Hallmark inflammatory response module score of B cells across HC, PD-Early, and PD-Late groups. Each group contains two biological replicates; therefore, all stage-associated patterns are exploratory.

Given that each group in GSE223138 contains only two biological replicates, all stage-associated findings in this section are exploratory and should be interpreted with caution regarding statistical power. The proportion of B cells showed a decreasing trend across the three PBMC groups, from 9.0% in the HC group, through 7.8% in the PD-Early group, to 6.1% in the PD-Late group (Figure 2A). This observation suggests a possible reduction in peripheral B-cell proportion during disease progression, but confirmation in larger stage-stratified cohorts is required. The dimensionality reduction and cell annotation results are shown in Figure 2 and Supplementary Figure S2.

The pseudobulk differential expression analysis suggested that the causal genes may exhibit distinct stage-associated expression patterns during PD progression. CD74, HLA-DRB1, and IL2RA showed expression differences detectable as early as the PD-Early stage and maintained a similar direction in PD-Late, and were therefore tentatively classified as the early-change pattern. As a representative early-change gene, CD74 showed an upward shift in single-cell expression distribution and pseudobulk mean expression in PD groups (Figure 2C). CR1 showed a later-stage expression difference and was tentatively classified as a late-change pattern, whereas BLK and BANK1 showed trends consistent with progressive changes. These patterns should be regarded as exploratory because of the small number of biological replicates. To provide complementary evidence, GSE194245 was used as an independent CD19 + B-cell dataset for subpopulation-level validation; however, because it lacks disease-stage information and differs in cell-isolation strategy, it was not treated as a formal merged-stage analysis. A cross-dataset comparison of the differential expression directions of the eight causal genes showed that five genes had fully concordant directions and one (CR1) showed partial concordance between the two datasets, supporting the biological plausibility of the MR-identified associations (Supplementary Table S4).

### 3.3 Fine localization and state transition of causal genes in B-cell subsets

**Figure 3:**
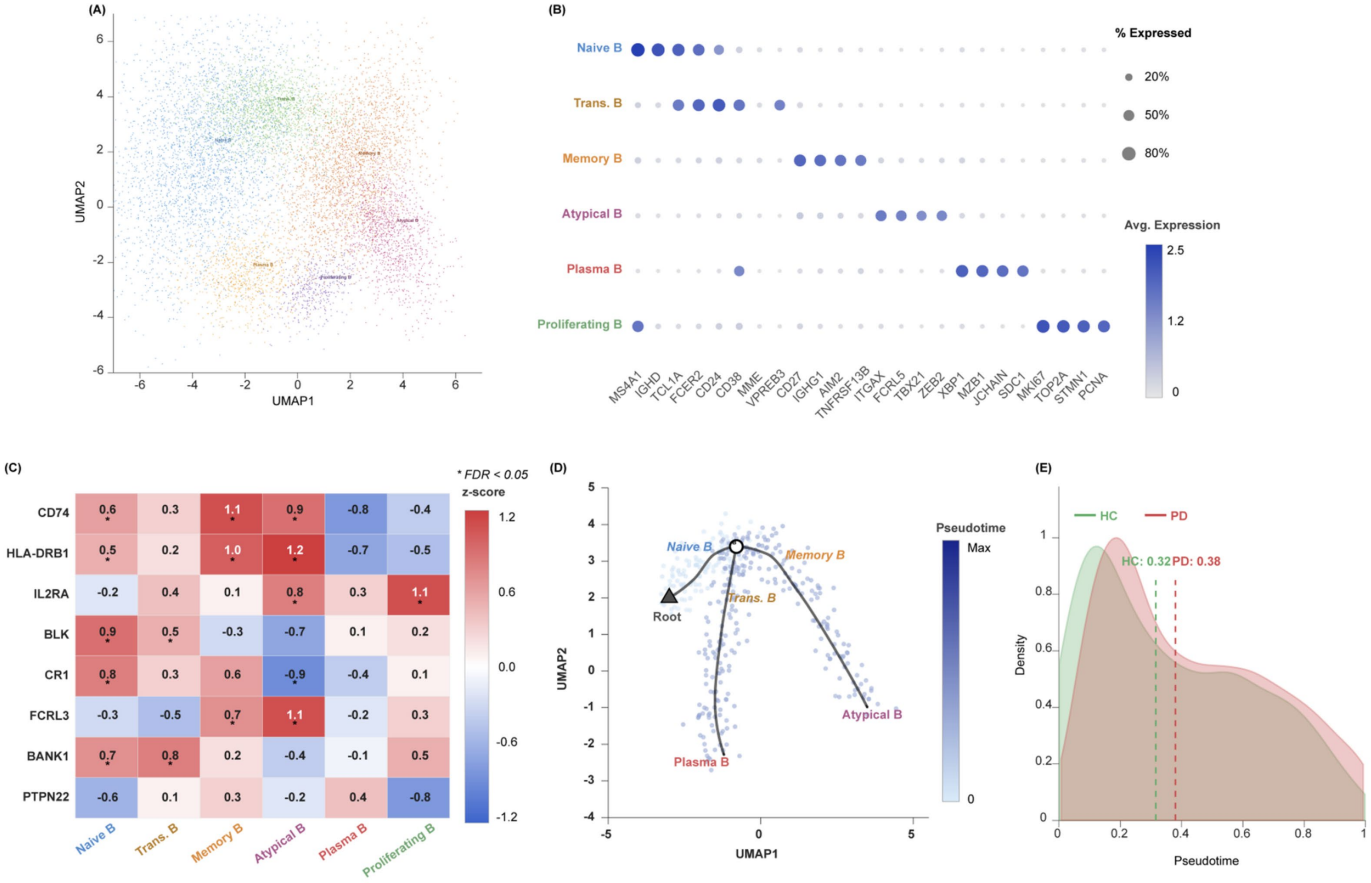
Fine localization and state transition analysis of causal genes in B-cell subpopulations. **(A)** UMAP visualization of 10,466 CD19^+^ sorted B cells from GSE194245, annotated into six subpopulations: Naive B, Transitional B, Memory B, Atypical B, Plasma B, and Proliferating B. **(B)** Dot plot of canonical marker gene expression across B-cell subpopulations. **(C)** Heatmap of PD-related differential expression (*z*-score) of 8 causal genes across B-cell subpopulations; asterisks indicate BH-adjusted FDR < 0.05. **(D)** Monocle pseudotime trajectory of B-cell differentiation with causal gene expression overlay. **(E)** Pseudotime density distribution of B cells in HC versus PD groups. Because BIN/BMem MR exposure categories do not map one-to-one to these six subpopulations, localization results should be interpreted as suggestive. *FDR < 0.05.

To further analyze the cellular heterogeneity of causal genes, independent cluster analysis was conducted on 10,466 CD19^+^ sorted B cells in GSE194245. The UMAP dimension reduction visualization identified a total of 6 different subpopulations of B cells, namely Naive B, Memory B, Transitional B, Atypical B, Proliferating B, and Plasma B, representing different differentiation and functional statuses (Figure 3A). The expression profiles of the classical marker genes of each subgroup of cells confirmed the reliability of annotation, as shown in the form of dot plots (Figure 3B).

The PD-related differential expression patterns of 8 causal genes in each B-cell subpopulation were presented as heatmaps, with asterisks indicating FDR < 0.05 (Figure 3C). The causal gene signals were mainly concentrated in Naive B and Atypical B subpopulations. Specifically, BLK and BANK1 showed PD-associated upregulation in Naive B cells, whereas CD74, HLA-DRB1, IL2RA, and CR1 showed differential expression patterns in Atypical B cells. However, given the resolution mismatch between broad MR exposure categories (BIN/BMem) and the six subpopulations identified here, these subpopulation-specific attributions should be regarded as suggestive enrichment patterns rather than definitive assignment of causal effects to individual B-cell states. MR-inferred effects for BIN may span Naive B and Transitional B cells, while effects for BMem may span Memory B and Atypical B cells.

Monocle3 pseudotime analysis suggested that five of the eight causal genes showed expression dynamics along the B-cell differentiation trajectory from naive to memory and effector states (Figure 3D). The pseudotime distribution of B cells in the PD group was shifted toward higher values compared with HC (median pseudotime: PD 0.38 vs. HC 0.32), suggesting a trend toward a more differentiated state in PD B cells, although this observation is based on a limited sample and should be interpreted with caution (Figure 3E).

### 3.4 Functional enrichment and immune inflammation pathway analysis

**Figure 4:**
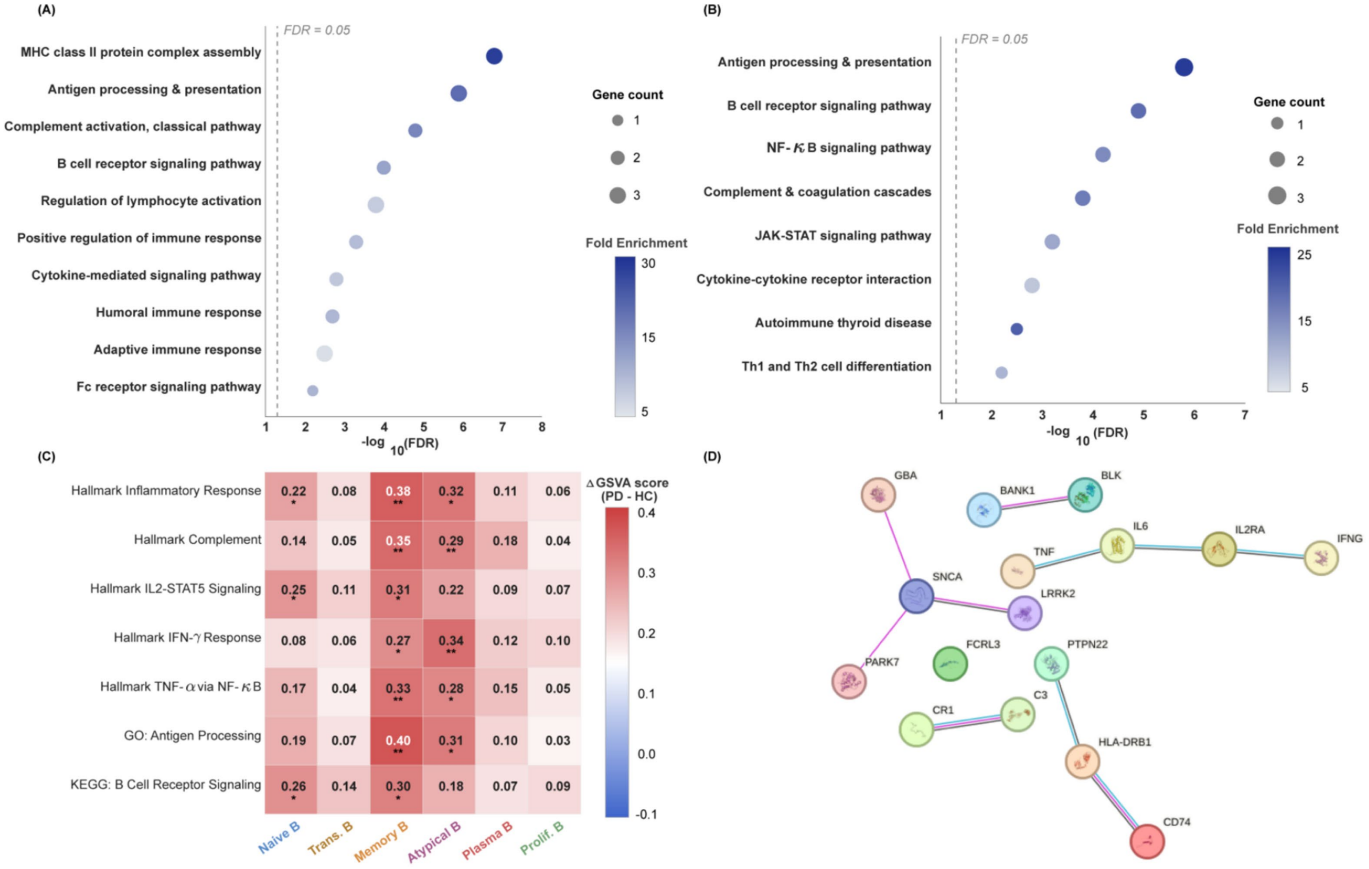
Functional enrichment and immune inflammation pathway analysis of causal genes. **(A)** GO biological process enrichment analysis of 8 high-confidence causal genes. **(B)** KEGG pathway enrichment analysis of 8 high-confidence causal genes. **(C)** GSVA heatmap of immune inflammation pathway activity changes (PD vs. HC) across B-cell subpopulations; asterisks indicate BH-adjusted FDR < 0.05. **(D)** Protein–protein interaction (PPI) network of causal genes, PD risk genes, and inflammatory mediators constructed from the STRING database (confidence score > 0.400). Key genes contributing to the enriched pathways are listed in Supplementary Table S3. *FDR < 0.05; **FDR < 0.01.

GO biological process enrichment analysis was conducted for the 8 high-confidence causal genes (Figure 4A). The most significantly enriched item was major histocompatibility complex (MHC) class II protein complex assembly, mainly involving CD74 and HLA-DRB1 (FDR = 1.2 × 10^−7^), followed by antigen processing and presentation (CD74, HLA-DRB1), classical pathway complement activation (CR1), B-cell receptor signaling (BLK, BANK1), and cytokine-mediated signaling (IL2RA) (all FDR < 0.05). These pathways suggest that the prioritized genes are involved in antigen presentation, B-cell activation, complement regulation, and cytokine signaling. In parallel, KEGG pathway enrichment highlighted antigen processing and presentation, B cell receptor signaling, JAK-STAT signaling, and complement and coagulation cascades (Figure 4B). The genes contributing to the key enriched pathways are summarized in Supplementary Table S3.

GSVA analysis further suggested PD-related changes in the activity of immune inflammation pathways in each B-cell subpopulation (Figure 4C). The Hallmark inflammatory response gene set, TNF-alpha signaling via NF-kB, complement-related pathways, and IL2-STAT5 signaling showed increased activity mainly in Memory B and Atypical B cells, while Naive B cells showed moderate increases in B-cell receptor signaling and IL2-STAT5 signaling. The Hallmark inflammatory response gene set includes inflammatory mediators and chemokines such as IL6, TNF, IL1B, CXCL10, CCL2, and related pathway genes; representative gene-set members used for GSVA are summarized in Supplementary Table S3. A protein–protein interaction network integrating the causal genes with PD risk genes and inflammatory mediators (STRING confidence > 0.400) further illustrated their connectivity (Figure 4D).

### 3.5 The intercellular communication network of peripheral B-cell immune inflammation

**Figure 5:**
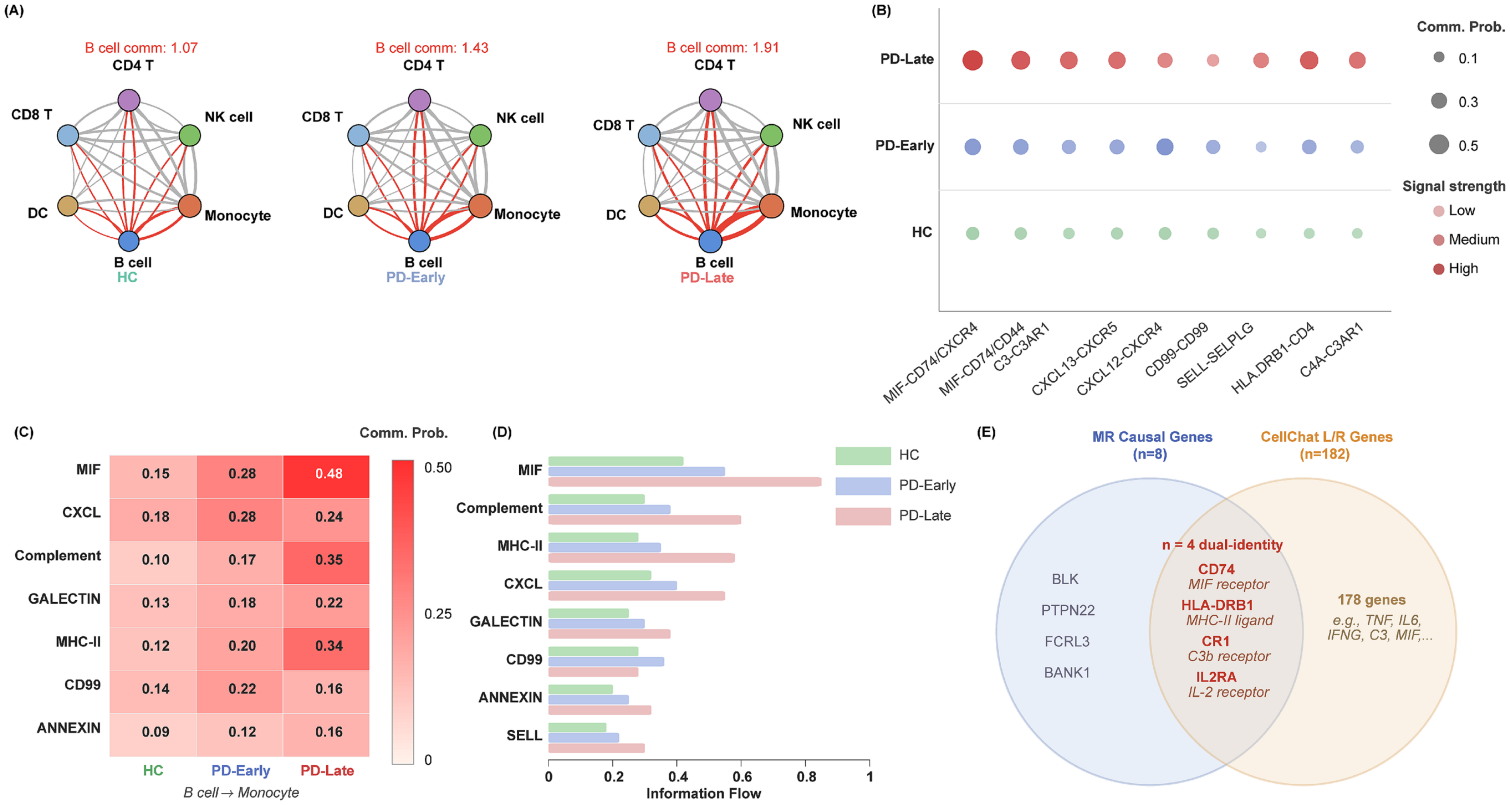
Computationally inferred peripheral B-cell intercellular communication network remodeling during Parkinson’s disease progression. **(A)** Circular plots of overall inferred intercellular communication intensity across HC, PD-Early, and PD-Late groups, with B-cell-related communication edges highlighted. **(B)** Dot plot of ligand-receptor pair communication probabilities across three groups, with signal strength indicated by dot size and color. **(C)** Quantitative assessment of inferred pathway-level communication probability from B cells to monocytes across three groups. **(D)** Global information flow comparison of signaling pathways across HC, PD-Early, and PD-Late groups. **(E)** Venn diagram showing the intersection of MR causal genes and CellChat ligand/receptor genes, identifying four dual-identity genes (CD74, HLA-DRB1, CR1, IL2RA). CellChat results are computational predictions based on gene expression and curated ligand-receptor interactions.

In comparing the circular diagrams of overall inferred intercellular communication intensity among the three groups, edges involving B cells are highlighted (Figure 5A). The inferred B-cell-related communication intensity showed an increasing trend from 1.07 in HC to 1.43 in PD-Early and 1.91 in PD-Late. Among these predicted interactions, the B cell-monocyte axis showed the strongest increase, consistent with the enrichment of antigen presentation and inflammatory signaling pathways. Because the communication scores were inferred from CellChat rather than experimentally measured, these findings should be interpreted as computational predictions.

At the ligand-receptor level, CellChat predicted that MIF-CD74/CXCR4 and MIF-CD74/CD44 had the strongest stage-associated increases among all ligand-receptor pairs (Figure 5B). Complement-related C3-C3AR1 and C4A-C3AR1 interactions, as well as antigen-presentation-related HLA-DRB1-CD4 interactions, also showed increased predicted communication. Not all pairs followed a monotonic increasing pattern; CXCL12-CXCR4 peaked in PD-Early and then partially decreased in PD-Late, and CD99-CD99 showed a similar non-monotonic trend. These communication predictions require orthogonal validation using experimental approaches such as co-culture assays, spatial transcriptomics, or protein-level measurements. Four MR causal genes (CD74, HLA-DRB1, CR1, IL2RA) were identified at the intersection of MR causal genes and CellChat ligand/receptor genes (Figure 5E).

At the pathway level, the MIF signaling pathway showed the greatest inferred enhancement from B cells to monocytes, rising from 0.15 in HC to 0.28 in PD-Early and 0.48 in PD-Late (Figure 5C), followed by the complement pathway (0.10–0.35) and the MHC-II pathway (0.12–0.34). Global information flow analysis indicated that the MIF pathway had the largest relative increase among all pathways from HC to PD-Late (Figure 5D). The CXCL and CD99 pathways showed non-monotonic patterns, peaking in PD-Early and partially declining in PD-Late.

### 3.6 Computational prioritization of druggable candidate targets

**Figure 6:**
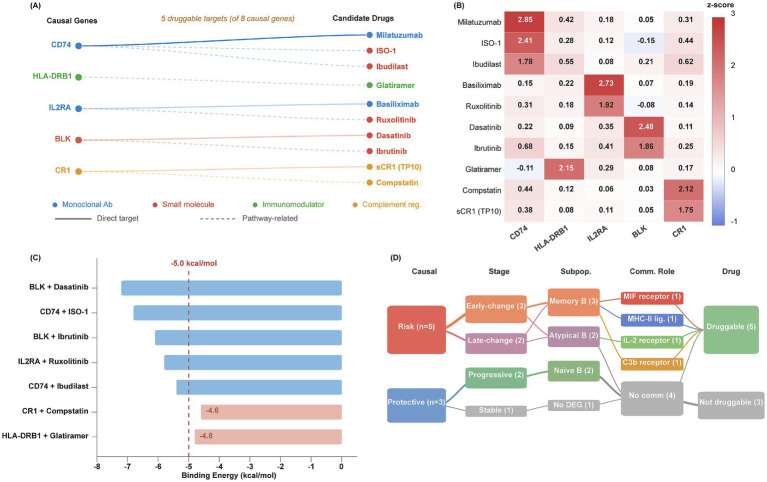
Druggable candidate target prediction and multi-dimensional evidence convergence. **(A)** Network mapping of 5 druggable causal genes to 10 candidate compounds via DGIdb, classified by drug type and targeting mode. **(B)** DSigDB enrichment heatmap (*z*-score) of 10 candidate compounds across 5 druggable causal genes. **(C)** AutoDock Vina molecular docking binding energies for selected small-molecule or peptide-like target pairs; dashed line indicates the −5.0 kcal/mol structural plausibility threshold. **(D)** Sankey plot tracing the five-dimensional evidence chain of each causal gene: MR causal direction, exploratory stage-associated pattern, subpopulation localization, predicted communication role, and druggability. Docking and druggability analyses are computational prioritization tools and do not validate pharmacological efficacy.

The druggability analysis aimed to evaluate whether existing drugs with established pharmacological annotations could serve as candidates for future repurposing studies in PD immunotherapy, based on the causal targets prioritized by MR analysis, rather than to validate known drug-target binding. Eight causal genes were compared with the DGIdb database, and five genes had druggability annotations (Figure 6A). Directly annotated drugs included Milatuzumab (anti-CD74 monoclonal antibody), Basiliximab (anti-IL2RA), Dasatinib (Src family kinase inhibitor covering BLK), and soluble CR1/TP10 (complement modulator). Pathway-related compounds included ISO-1 and Ibudilast (MIF inhibitors upstream of CD74), Glatiramer (MHC-II competitor), and Compstatin (C3 inhibitor related to the CR1 pathway). These results nominate candidate compounds for future validation but do not establish therapeutic efficacy in PD (Supplementary Table S2).

DSigDB enrichment analysis of 10 candidate compounds for 5 druggable causal genes suggested enrichment patterns linking Milatuzumab and ISO-1 with CD74, Basiliximab with IL2RA, Dasatinib with BLK, and Compstatin/sCR1 with CR1 (Figure 6B). Because the binding of target-specific monoclonal antibodies to their designed targets is expected, this analysis was interpreted as druggability annotation rather than novel pharmacological validation. Molecular docking was therefore emphasized only for selected small-molecule or peptide-like interactions as a structural plausibility assessment. Dasatinib-BLK (−7.2 kcal/mol) and ISO-1-CD74 (−6.8 kcal/mol) showed favorable docking scores (Figure 6C), while these values cannot substitute for experimental binding or functional assays. Ibudilast was retained as a hypothesis-generating repurposing candidate because of its MIF-related mechanism and prior neurological safety information, but no therapeutic conclusion for PD can be drawn from the present computational analysis.

The Sankey plot illustrates the evidence chain of individual causal genes across five dimensions: MR causal direction, exploratory stage-associated pattern, subpopulation enrichment, predicted communication role, and druggability (Figure 6D). CD74 showed the most complete convergence evidence, spanning risk-increasing MR effect, early-change expression pattern, Memory/Atypical B-cell enrichment, MIF receptor function, and targetability by ISO-1 or Ibudilast. IL2RA showed convergence across risk-increasing MR effect, early-change pattern, Atypical B-cell enrichment, IL-2 receptor function, and Basiliximab annotation. CR1 represented a protective gene linked to complement regulation and candidate complement-modulating strategies. These convergent signals prioritize genes and compounds for future validation rather than confirming therapeutic efficacy.

## 4 Discussion

Our computational integration suggests that peripheral B cells, through genetically predicted expression changes and computationally inferred intercellular communication remodeling, may represent an underappreciated component of the peripheral immune landscape in PD. The high-confidence MR causal genes prioritized through colocalization and replication were not uniformly distributed across B-cell states; instead, they showed suggestive enrichment in Naive B and Atypical B subpopulations. These findings provide a hypothesis-generating framework linking B-cell genetic regulation, transcriptomic alteration, communication inference, and druggability prioritization.

The previous peripheral blood single-cell studies have systematically characterized the phenotypic changes of B cells in PD patients, which included an increased proportion of memory B cells, a decreased proportion of immature B cells, and the upregulation of antigen presenting related genes (25). The single-cell atlas of cerebrospinal fluid immune cells also showed significant parallelism between peripheral and central immune changes (26). However, these descriptive findings have always been challenged by the basic epistemological question: Do these B-cell phenotypic variations represent the pathogenic factors in PD, or do they represent the immune concomitant neurodegenerative processes? This paper substantially mitigated the confounding issue by using the genetic instrumental variables in MR to identify the set of causal genes underlying the aforementioned B-cell phenotypic variations and providing a genetic mechanism for the activation of the MHC-II antigen presentation pathway and the variation in B-cell subsets, rather than just the description of the phenomenon. It is notable that the contradictory phenomenon mentioned in the introduction that *α* -synuclein-specific autoantibodies decrease in the prodromal stage of PD has a potential explanation within the causal framework of this study: The early-change causal genes may contribute to the activation of Atypical B in PD-Early, and Atypical B is considered to have the functional characteristics of reduced antibody production efficiency but enhanced secretion of inflammatory cytokines in autoimmune studies. This subpopulation variation may be exactly the key to explaining the seemingly contradictory fact that, during the early stage of PD, B cell activation is concomitant with decreased levels of specific antibodies. Recent research suggested that the regulation of gene splicing with genetic variation is highly specific for cell type (27). This not only validates the rationality of the methodology of using B cell subtype-level sc-eQTL data in this research, but it also implies that the causal genes may regulate the functional state of B cells via splicing isomer transformation rather than expression level variation - although this remains beyond the scope of data validation, it hints at an issue worth exploring for potential research into the mechanism.

At the methodological level, the SC-EQTL-driven MR studies have achieved significant advancements in the field of the causal relationship between immune cells and PD, and a wide coverage framework for the parallel analysis of multiple types of immune cells is generally used. Although the integration analysis of multi-omics data identified genetic signals for PD in different types of peripheral immune cells, B cells were only included as one type of immune cells in the entire category (28). The Bidirectional MR Design System explored the causal relationship in both directions between immune cell phenotypes and PD, but it did not explore the functional localization of causal genes at the subpopulation level (15). Broad-spectrum screening of immune cells and neurodegenerative diseases provides a macroscopic reference for understanding the immune-degenerative disease axis, but its resolution is insufficient to capture signal heterogeneity within subpopulations (29). MR analysis of the levels of immune mediators, in combination with the causal information from the cytokine dimension, did not have functional links to the subpopulations of the cells that produce the cytokines (30). The possible conclusions of “weak association between B cells and PD” or “no significant association” at the overall B-cell level that the above-mentioned research might have drawn, this study reveals the underlying reasons: When the upregulation of risk genes in a certain subpopulation and that of protective genes in the same subpopulation are mixed and analyzed, the signals in opposite directions cancel each other out at the overall level, resulting in the dilution or even complete masking of the true subgroup-specific causal effect. Cross-disease sc-eQTL integration analysis has verified that the gene regulatory effect of genetic variation is highly dependent on the disease state and cell environment (31). This gives us a theoretical basis for the dynamic pattern of stages that has been discovered in the current study—the same causal gene has different intensities of effect at different stages of the disease. It might precisely be the expression of the effect of the disease state-dependent eQTL effect in the time dimension. The single cell type deep paradigm demonstrated in the current research, focusing on B cells for causal screening, localization of subpopulations, state transition, communication network, and the entire chain of drug target analysis, not only compensates for the inherent resolution problem of the wide coverage paradigm but also offers an applicable framework for other extremely heterogeneous cell populations of the immune system, such as T cell subsets and monocyte subsets.

This study directly analyzed immune inflammatory characteristics of peripheral blood B cells. The relationship between peripheral immune inflammation and CNS neuroinflammation should be interpreted within the broader framework of peripheral-central immune interaction rather than as direct evidence of CNS involvement. The B cell-monocyte MIF-CD74 communication axis predicted in this study increased across PD stages. MIF has been reported to participate in peripheral immune responses and may also affect microglial signaling through CD74 under certain conditions. Although speculative, this cross-compartment expression of CD74 raises the possibility that the MIF-CD74 axis may represent a molecular link between peripheral immune activation and CNS neuroinflammatory processes. This hypothesis requires direct experimental investigation using approaches such as blood–brain barrier models, CSF proteomics, brain tissue datasets, or functional co-culture systems. Similarly, CR1 may connect peripheral complement regulation with inflammatory pathways, but its role in PD-related neural injury remains to be experimentally tested.

In terms of translational implications, the causal targets prioritized by this study nominate candidate immune intervention hypotheses that differ from classical alpha-synuclein-targeting strategies. Ibudilast, which has neurological safety information and is related to MIF pathway modulation, emerges as a hypothesis-generating repurposing candidate for CD74-related immunomodulation. However, this prediction is based entirely on public-data integration, druggability annotation, and molecular docking. Dedicated preclinical and clinical studies are required before any therapeutic conclusion can be drawn. Therefore, the druggability results should be interpreted as prioritization for future testing rather than as validation of treatment efficacy.

Several limitations should be emphasized. First, the entire study relies on secondary analyses of public datasets without direct experimental, animal, protein-level, or clinical validation. The identified causal genes, communication pathways, and candidate compounds should therefore be viewed as testable hypotheses. Second, the stage-associated analysis in GSE223138 is limited by the very small number of biological replicates (*n* = 2 per group). Although pseudobulk aggregation was used to avoid single-cell pseudo-replication, statistical power remains limited, and power analyses of multi-sample scRNA-seq studies indicate that increasing the number of biological replicates is often more important than increasing cell numbers alone (32). Therefore, true biological differences or false-positive findings cannot be fully excluded. Third, the datasets used in this study were generated from different cohorts, laboratories, sequencing platforms, population backgrounds, and processing protocols. OneK1K sc-eQTL data were derived from healthy donors, whereas regulatory effects may be context-dependent under disease conditions, a limitation also emphasized by single-cell eQTL studies of immune regulation (10). Fourth, the MR exposure categories BIN and BMem do not map one-to-one to the six B-cell subpopulations annotated in GSE194245; therefore, subpopulation-specific localization should be regarded as suggestive rather than definitive. Fifth, non-significant reverse MR results should be interpreted cautiously because reverse MR using gene expression as the outcome may have limited statistical power and MR assumptions can be affected by pleiotropy and directionality issues (33). Sixth, CellChat inference was based on one computational tool and curated ligand-receptor databases; benchmark studies suggest that different tools—such as CellPhoneDB and NicheNet—may return partially distinct interaction sets and that combining complementary methods can improve confidence in inferred interactions (23, 24). Seventh, druggability prediction and molecular docking provide computational prioritization and structural plausibility only and cannot establish pharmacological efficacy or clinical utility. Finally, although GSE194245 includes paired BCR-seq data, this information was not incorporated because the present framework focused on expression-mediated genetic effects, subpopulation localization, and ligand-receptor communication. Prior PD B-cell studies have shown that BCR-seq can reveal clonal and isotype-related features of peripheral B cells (7); future integration of BCR repertoire features may clarify whether these candidate genes are associated with antigen-driven clonal expansion or antigen specificity in PD.

## 5 Conclusion

In summary, this computational integration of B-cell sc-eQTL-based MR and public single-cell transcriptomic datasets prioritized CD74, IL2RA, and CR1 as candidate B-cell-related genes potentially linked to PD immune dysregulation. Exploratory analyses suggested stage-associated expression patterns, subpopulation enrichment, and predicted intercellular communication changes, particularly involving the MIF-CD74 axis. Druggability annotation and molecular docking nominated candidate repurposing hypotheses but did not validate therapeutic efficacy. Given the reliance on public datasets and computational inference, these findings should be regarded as hypothesis-generating. Larger longitudinal cohorts, functional experiments, protein-level assays, and clinical validation will be required to determine their biological and therapeutic relevance.

## Data Availability

Publicly available datasets were analyzed in this study. This data can be found here: GEO: GSE223138 (https://www.ncbi.nlm.nih.gov/geo/query/acc.cgi?acc=GSE223138); GSE194245 (https://www.ncbi.nlm.nih.gov/geo/query/acc.cgi?acc=GSE194245). OneK1K eQTL: https://onek1k.org. DICE eQTL: https://dice-database.org. IPDGC PD GWAS: https://pdgenetics.org. FinnGen: https://www.finngen.fi.
