# Supplementary material for: Peripheral B cell immune dysregulation genetically contributes to stage-dependent neuroinflammation and identifies priority therapeutic targets in Parkinson’s disease: a computational integration of Mendelian randomization and single-cell transcriptomics
Source: Front Med (Lausanne). 2026 Jun 9;13:1853077. doi: 10.3389/fmed.2026.1853077 (PMC13286758; doi:10.3389/fmed.2026.1853077)
Supplement: Supplementary file 1 [file Data_Sheet_1.docx]

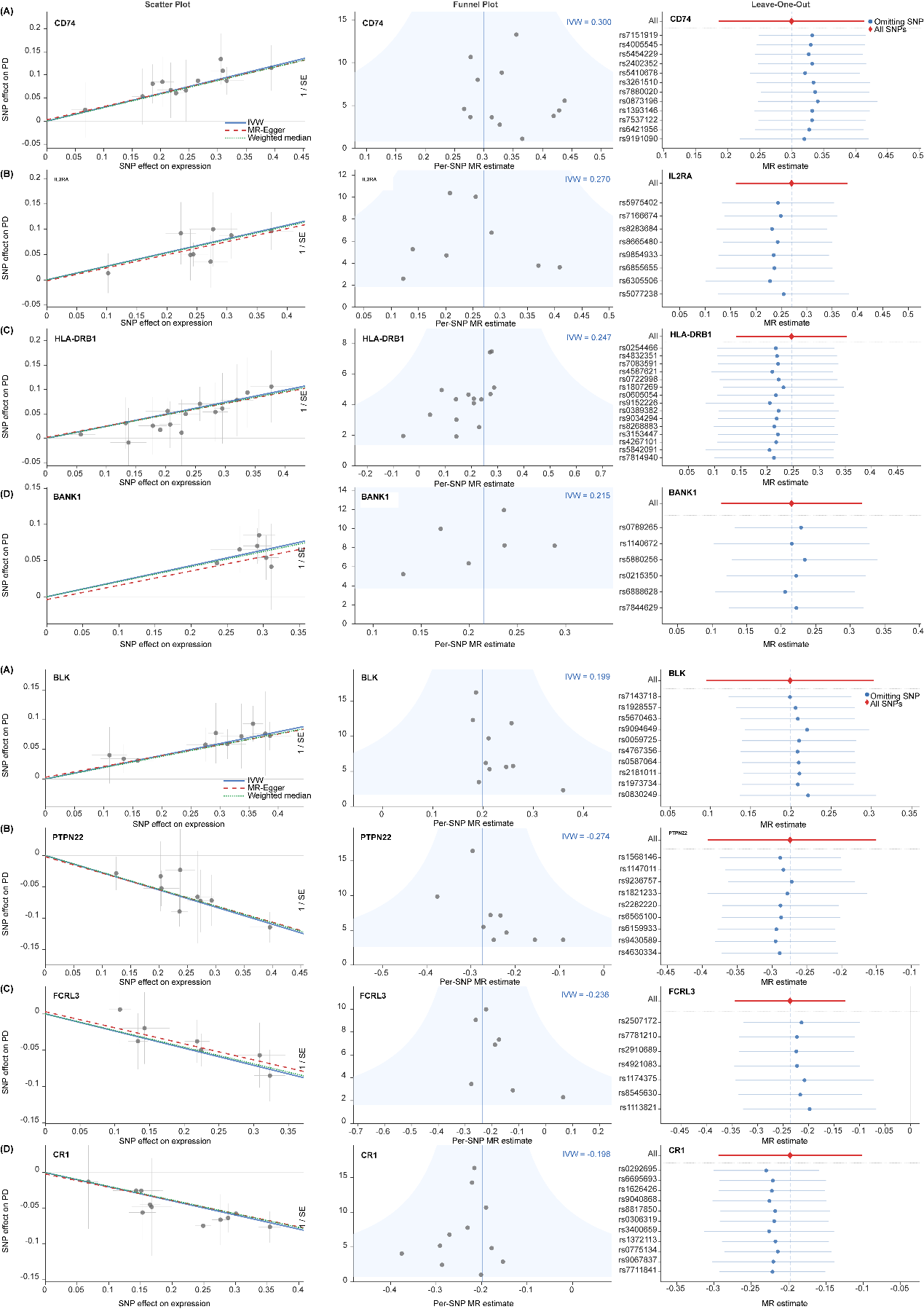


**Figure S1.** **Sensitivity analysis for Mendelian randomization of 8 high-confidence causal genes.**

For each gene (page 1 A–D: CD74, IL2RA, HLA-DRB1, BANK1; page 2 A–D: BLK, PTPN22, FCRL3, CR1): scatter plot of per-SNP effect estimates with fitted lines for IVW, MR-Egger, and weighted median methods (left); funnel plot of per-SNP MR estimates (center); leave-one-out forest plot (right).


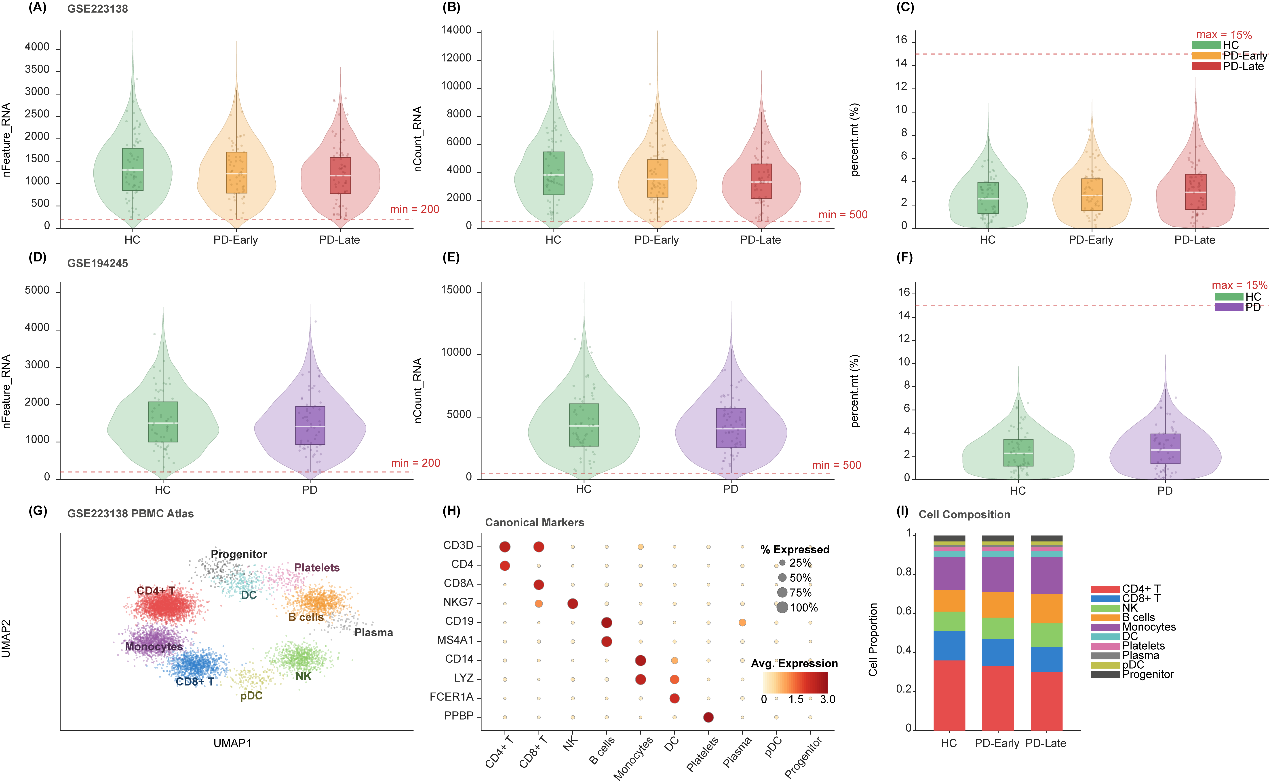


**Figure S2.** **Quality control and cell-type annotation of scRNA-seq datasets. Sample sizes: n = 2 per group for GSE223138; n = 8 PD and 6 HC for GSE194245.**

(A–C) Quality control metrics for GSE223138: number of detected genes (nFeature_RNA), UMI counts (nCount_RNA), and mitochondrial gene percentage across HC, PD-Early, and PD-Late groups.

(D–F) Quality control metrics for GSE194245 across HC and PD groups.

(G) UMAP visualization of the full PBMC dataset in GSE223138 with cell-type annotations.

(H) Dot plot of canonical marker gene expression for major PBMC cell types.

(I) Cell-type composition across HC, PD-Early, and PD-Late groups.
