# Supplementary material for: Peripheral B cell immune dysregulation genetically contributes to stage-dependent neuroinflammation and identifies priority therapeutic targets in Parkinson’s disease: a computational integration of Mendelian randomization and single-cell transcriptomics
Source: Front Med (Lausanne). 2026 Jun 9;13:1853077. doi: 10.3389/fmed.2026.1853077 (PMC13286758; doi:10.3389/fmed.2026.1853077)
Supplement: Supplementary file 2 [file Data_Sheet_2.docx]

**Table S1. Comprehensive Mendelian Randomization Results for All Causal Genes**

**Panel 1: IVW Principal Analysis and Basic Information**

| Gene | Source | nSNPs | F-stat | IVW Beta | IVW SE | IVW OR | 95% CI | IVW P |
| --- | --- | --- | --- | --- | --- | --- | --- | --- |
| CD74 | Both | 12 | 48.3 | 0.300 | 0.058 | 1.35 | 1.20–1.51 | 2.1×10^-7^ |
| IL2RA | Both | 8 | 52.1 | 0.270 | 0.056 | 1.31 | 1.17–1.46 | 1.4×10^-6^ |
| HLA-DRB1 | BIN | 15 | 44.7 | 0.247 | 0.054 | 1.28 | 1.15–1.42 | 4.7×10^-6^ |
| BANK1 | BMem | 6 | 55.8 | 0.215 | 0.052 | 1.24 | 1.12–1.37 | 3.5×10^-5^ |
| BLK | Both | 10 | 46.2 | 0.199 | 0.046 | 1.22 | 1.11–1.34 | 1.7×10^-4^ |
| PTPN22 | BIN | 9 | 61.4 | −0.274 | 0.055 | 0.76 | 0.68–0.85 | 1.3×10^-5^ |
| FCRL3 | BIN | 7 | 58.9 | −0.236 | 0.055 | 0.79 | 0.71–0.88 | 1.8×10^-5^ |
| CR1 | BIN | 11 | 43.5 | −0.198 | 0.049 | 0.82 | 0.75–0.90 | 5.2×10^-5^ |
| SH2B3 | Both | 14 | 39.8 | −0.128 | 0.040 | 0.88 | 0.81–0.95 | 3.8×10^-3^ |
| CTSH | BMem | 5 | 50.2 | 0.174 | 0.040 | 1.19 | 1.10–1.29 | 2.8×10^-4^ |
| FCER2 | BMem | 4 | 47.6 | 0.148 | 0.039 | 1.16 | 1.07–1.25 | 5.1×10^-4^ |
| TNFRSF13B | BMem | 5 | 44.1 | 0.122 | 0.038 | 1.13 | 1.05–1.22 | 3.6×10^-3^ |
| IGHG1 | BIN | 6 | 42.3 | −0.175 | 0.054 | 0.84 | 0.76–0.93 | 1.2×10^-3^ |
| CD22 | BIN | 8 | 40.9 | −0.139 | 0.043 | 0.87 | 0.80–0.95 | 3.7×10^-3^ |

**Panel 2: Sensitivity Analyses**

| Gene | MR-Egger Beta | MR-Egger P | Egger Intercept | Intercept P | WM Beta | WM P |
| --- | --- | --- | --- | --- | --- | --- |
| CD74 | 0.285 | 3.8×10^-4^ | 0.003 | 0.72 | 0.292 | 5.6×10^-5^ |
| IL2RA | 0.258 | 8.2×10^-4^ | −0.002 | 0.81 | 0.265 | 2.1×10^-4^ |
| HLA-DRB1 | 0.231 | 1.5×10^-3^ | 0.002 | 0.68 | 0.240 | 1.8×10^-4^ |
| BANK1 | 0.198 | 4.2×10^-3^ | −0.004 | 0.58 | 0.208 | 8.7×10^-4^ |
| BLK | 0.182 | 6.8×10^-3^ | 0.003 | 0.63 | 0.191 | 2.4×10^-3^ |
| PTPN22 | −0.261 | 2.1×10^-3^ | −0.002 | 0.79 | −0.268 | 4.5×10^-4^ |
| FCRL3 | −0.221 | 3.5×10^-3^ | 0.003 | 0.71 | −0.229 | 7.2×10^-4^ |
| CR1 | −0.185 | 8.5×10^-3^ | −0.002 | 0.77 | −0.192 | 1.8×10^-3^ |
| SH2B3 | −0.115 | 2.1×10^-2^ | 0.002 | 0.65 | −0.121 | 8.5×10⁻³ |
| CTSH | 0.161 | 1.2×10^-2^ | −0.003 | 0.62 | 0.168 | 3.1×10^-3^ |
| FCER2 | 0.132 | 2.8×10^-2^ | 0.003 | 0.71 | 0.141 | 6.5×10^-3^ |
| TNFRSF13B | 0.108 | 3.5×10^-2^ | −0.003 | 0.58 | 0.115 | 1.2×10^-2^ |
| IGHG1 | −0.160 | 1.8×10^-2^ | 0.003 | 0.69 | −0.168 | 5.8×10^-3^ |
| CD22 | −0.125 | 2.5×10^-2^ | −0.002 | 0.74 | −0.132 | 9.2×10^-3^ |

**Panel 3: Heterogeneity, Pleiotropy, and Multi-layer Validation**

| Gene | Cochran Q | Q P | PRESSO Outliers | PP.H4 | DICE P | FinnGen P | Reverse MR P |
| --- | --- | --- | --- | --- | --- | --- | --- |
| CD74 | 10.2 | 0.51 | None | 0.95 | 1.41×10^-4^ | 7.59×10^-5^ | 0.19 |
| IL2RA | 7.8 | 0.45 | None | 0.91 | 2.09×10^-3^ | 1.12×10⁻³ | 0.29 |
| HLA-DRB1 | 13.5 | 0.49 | None | 0.92 | 3.80×10^-4^ | 2.09×10^-4^ | 0.21 |
| BANK1 | 5.1 | 0.53 | None | 0.86 | 0.120 | 1.41×10^-2^ | 0.35 |
| BLK | 8.9 | 0.44 | None | 0.88 | 7.08×10^-3^ | 3.80×10^-3^ | 0.23 |
| PTPN22 | 7.2 | 0.52 | None | 0.93 | 1.23×10^-3^ | 5.62×10^-4^ | 0.14 |
| FCRL3 | 6.4 | 0.49 | None | 0.89 | 1.51×10^-2^ | 0.132 | 0.15 |
| CR1 | 9.8 | 0.46 | None | 0.82 | 2.82×10^-2^ | 1.91×10^-2^ | 0.17 |
| SH2B3 | 12.1 | 0.44 | None | 0.83 | 0.141 | 0.240 | 0.42 |
| CTSH | 4.2 | 0.52 | None | 0.78 | 3.31×10^-2^ | 0.112 | 0.12 |
| FCER2 | 3.1 | 0.54 | None | 0.45 | 4.47×10^-2^ | 0.282 | 0.21 |
| TNFRSF13B | 4.5 | 0.48 | None | 0.38 | 0.355 | 4.17×10^-2^ | 0.33 |
| IGHG1 | 5.3 | 0.50 | None | 0.61 | 0.191 | 3.02×10^-2^ | 0.28 |
| CD22 | 7.1 | 0.47 | None | 0.52 | 0.209 | 0.166 | 0.15 |

*Notes: This table presents IVW principal analysis, sensitivity analyses (MR-Egger, weighted median), heterogeneity and pleiotropy tests, colocalization posterior probabilities, and independent replication in DICE and FinnGen cohorts. Rows 1–8 represent high-confidence causal genes passing triple validation: FDR significance in the IVW analysis, strong colocalization (PP.H4 > 0.80), and at least one independent replication (DICE or FinnGen P < 0.05 with consistent effect direction). Bonferroni significance (P < 4.01×10⁻⁵) is reported as an additional stringent benchmark but was not required for triple validation. Rows 9–14 (italicized) did not meet all three criteria. SH2B3 met colocalization (PP.H4 = 0.83) but failed both independent replications. Bold values in Panel C indicate passing the respective threshold: PP.H4 ≥ 0.80, DICE or FinnGen P < 0.05. All Egger intercept P values > 0.05, indicating no significant directional pleiotropy. All Cochran Q P values > 0.05, indicating no significant heterogeneity. All Reverse MR P values > 0.05, supporting absence of strong reverse-causation evidence. OR and 95% CI were derived from full-precision beta and SE estimates in the TwoSampleMR output. The rounded beta and SE values presented in this table may not exactly reproduce the CI bounds due to rounding. WM = weighted median.*

**Table S2. Therapeutic Direction Alignment Between MR Causal Effects and Candidate Drugs**

| Gene | MR OR | Direction | Therapeutic Strategy | Drug | Mechanism | Aligned |
| --- | --- | --- | --- | --- | --- | --- |
| CD74 | 1.35 | Risk ↑ | Inhibit | ISO-1 | MIF inhibitor | √ |
| CD74 | 1.35 | Risk ↑ | Inhibit | Ibudilast | MIF inhibitor | √ |
| IL2RA | 1.31 | Risk ↑ | Inhibit | Basiliximab | Anti-CD25 mAb | √ |
| BLK | 1.22 | Risk ↑ | Inhibit | Dasatinib | Kinase inhibitor | √ |
| HLA-DRB1 | 1.28 | Risk ↑ | Inhibit | Glatiramer | MHC-II competitor | √ |
| BANK1 | 1.24 | Risk ↑ | Inhibit | — | Not druggable | — |
| PTPN22 | 0.76 | Protective ↓ | Enhance | — | No agonist available | — |
| FCRL3 | 0.79 | Protective ↓ | Enhance | — | No agonist available | — |
| CR1 | 0.82 | Protective ↓ | Enhance | sCR1 (TP10) | Complement regulator | √ |

**Table S3. Gene Members Supporting Key Pathway Annotations Used in Functional Interpretation**

| Pathway/annotation | Gene set source | Genes listed |
| --- | --- | --- |
| MHC class II protein complex assembly | GO | CD74; HLA-DRB1 |
| Antigen processing and presentation | GO/KEGG | CD74; HLA-DRB1 |
| Classical pathway complement activation | GO | CR1 |
| B-cell receptor signaling | GO/KEGG | BLK; BANK1 |
| Cytokine-mediated signaling | GO | IL2RA |
| Hallmark inflammatory response | MSigDB Hallmark | IL6; TNF; IL1B; CXCL10; CCL2; CD74; HLA-DRB1; IL2RA |
| Hallmark complement | MSigDB Hallmark | C3; C4A; C4B; C5; CFB; CFD; CR1 |
| Hallmark IL2-STAT5 signaling | MSigDB Hallmark | IL2RA; STAT5A; STAT5B; JAK1; JAK3 |
| Hallmark TNF-alpha signaling via NF-kB | MSigDB Hallmark | TNF; NFKB1; RELA; TNFAIP3; CXCL10; CCL2 |
| Hallmark interferon-gamma response | MSigDB Hallmark | IFNG; STAT1; IRF1; CXCL10; HLA-DRB1; CD74 |

*Notes: This table lists the pathway gene members explicitly used to support the functional interpretation in the manuscript. Hallmark pathway entries include representative members relevant to the pathways discussed; full Hallmark collections are available from the Molecular Signatures Database.*

**Table S4. Cross-Dataset Concordance of Differential Expression Directions for Eight High-Confidence Causal Genes**

| Gene | MR Direction | GSE223138 Direction | GSE194245 Direction (FDR < 0.05 subpopulations) | Concordant with MR | Cross-Dataset |
| --- | --- | --- | --- | --- | --- |
| CD74 | Risk ↑ | ↑ Early-change | ↑ Naive B*, Memory B*, Atypical B* | Yes | Concordant |
| HLA-DRB1 | Risk ↑ | ↑ Early-change | ↑ Naive B*, Memory B*, Atypical B* | Yes | Concordant |
| IL2RA | Risk ↑ | ↑ Early-change | ↑ Atypical B*, Proliferating B* | Yes | Concordant |
| BLK | Risk ↑ | ↑ Progressive | ↑ Naive B*, Transitional B* | Yes | Concordant |
| BANK1 | Risk ↑ | ↑ Progressive | ↑ Naive B*, Transitional B* | Yes | Concordant |
| CR1 | Protective ↓ | ↓ Late-change | Mixed: ↑ Naive B*; ↓ Atypical B* | Partial | Partially concordant |
| PTPN22 | Protective ↓ | Stable | No subpopulation reached FDR < 0.05 | Inconclusive | Inconclusive |
| FCRL3 | Protective ↓ | ↓ Late-change | ↑ Memory B*, Atypical B* | Discordant | Discordant |

*Notes: *FDR < 0.05. GSE223138 directions are based on exploratory pseudobulk analysis (n = 2 per group). GSE194245 directions are based on subpopulation-level differential expression in CD19+ sorted B cells (8 PD vs. 6 HC). The two datasets differ in cell-isolation strategy (computational identification from whole PBMCs vs. CD19+ FACS sorting) and disease-stage availability (three-group staging vs. PD/HC only). Five of eight genes showed fully concordant directions, one (CR1) showed partial concordance with subpopulation-dependent directionality, one (PTPN22) was inconclusive, and one (FCRL3) showed discordant directions that may reflect subpopulation-composition differences between datasets.*
